# Brain and behavior changes associated with an abbreviated 4‐week mindfulness‐based stress reduction course in back pain patients

**DOI:** 10.1002/brb3.443

**Published:** 2016-02-16

**Authors:** B. Blair Braden, Teri B. Pipe, Ryan Smith, Tyler K. Glaspy, Brandon R. Deatherage, Leslie C. Baxter

**Affiliations:** ^1^ Department of NeuroimagingBarrow Neurological InstituteSt. Joseph's Hospital and Medical CenterPhoenixArizona; ^2^ College of Nursing and Health InnovationArizona State UniversityPhoenixArizona; ^3^ Departments of Psychiatry and PsychologyUniversity of ArizonaTucsonArizona

**Keywords:** Anxiety, back pain, depression, frontal lobe, functional magnetic resonance imaging, mindfulness, stress

## Abstract

**Introduction:**

Mindfulness‐based stress reduction (MBSR) reduces depression, anxiety, and pain for people suffering from a variety of illnesses, and there is a growing need to understand the neurobiological networks implicated in self‐reported psychological change as a result of training. Combining complementary and alternative treatments such as MBSR with other therapies is helpful; however, the time commitment of the traditional 8‐week course may impede accessibility. This pilot study aimed to (1) determine if an abbreviated MBSR course improves symptoms in chronic back pain patients and (2) examine the neural and behavioral correlates of MBSR treatment.

**Methods:**

Participants were assigned to 4 weeks of weekly MBSR training (*n* = 12) or a control group (stress reduction reading; *n* = 11). Self‐report ratings and task‐based functional MRI were obtained prior to, and after, MBSR training, or at a yoked time point in the control group.

**Results:**

While both groups showed significant improvement in total depression symptoms, only the MBSR group significantly improved in back pain and somatic‐affective depression symptoms. The MBSR group also uniquely showed significant increases in regional frontal lobe hemodynamic activity associated with gaining awareness to changes in one's emotional state.

**Conclusions:**

An abbreviated MBSR course may be an effective complementary intervention that specifically improves back pain symptoms and frontal lobe regulation of emotional awareness, while the traditional 8‐week course may be necessary to detect unique improvements in total anxiety and cognitive aspects of depression.

## Introduction

Back pain is one of the most common health problems, with an estimated lifetime global prevalence of 40% (Hoy et al. 2012). Mood disturbances, particularly depression and anxiety, are both risk factors for, and the result of, chronic back pain (Linton 2000; Rush et al. 2000; Gerhardt et al. 2011). Approximately 50% of chronic back pain patients experience concomitant depression, and there is a clear positive relationship in severity between the two conditions (Rush et al. 2000). In many cases, treatment outlook for chronic back pain is grim. The effectiveness of most analgesic pharmaceutical interventions is low (Machado et al. 2009) and the risk‐to‐benefit ratio for many surgical interventions is unfavorable (Chou et al. 2009). Many patients who experience chronic syndromes such as back pain, depression, or anxiety are combining integrative therapies with allopathic medicine. Mindfulness‐based stress reduction (MBSR) is a structured training program that aims to provide adaptive coping, focused attention, and cognitive restructuring skills to distressed populations (Kabat‐Zinn 1990). Mindfulness‐based stress reduction intervention studies in back pain patients show demonstrably favorable outcomes on pain severity, functional limitations due to pain, acceptance of chronic pain, physical functioning, and depression (Morone et al. 2008; Rosenzweig et al. 2010; Schutze et al. 2014). The psychological or physical mechanisms underlying MBSR's effectiveness have not been completely explained in terms of any single factor; it is likely that training instead provides improved functioning in a number of ways, as favorable outcomes have been observed on emotion‐related symptoms across diverse ailments such as anxiety disorders (Goldin and Gross 2010; Vollestad et al. 2011; Asmaee Majid et al. 2012), cancer (Lengacher et al. 2009; Johns et al. 2015), neurosurgery (Joo et al. 2010), post‐traumatic stress disorder (PTSD) and suicidality in veterans (Omidi et al. 2013; Serpa et al. 2014), and adult survivors of childhood sexual abuse (Kimbrough et al. 2010).

In a recent meta‐analysis across diverse populations, emotional and cognitive reactivity were identified as the strongest and the most consistent psychological constructs to mediate mindfulness‐related benefits (Gu et al. 2015). These psychological processes may correspond to brain regions implicated in emotion regulation, including the ventrolateral and dorsomedial prefrontal cortices (vlPFC and dmPFC), anterior insula (AI), anterior cingulate cortex (ACC), as well as subcortical regions (Ochsner and Gross 2005; Ochsner et al. 2012). For example, patients with depression show functional changes in brain regions associated with both appraising the emotional significance of one's situation (subgenual ACC; sgACC) and with perception of the bodily emotional reactions that those appraisals provoke (AI; Price and Drevets 2012). In both depression and anxiety studies, there is evidence that MBSR training modulates blood oxygen level‐dependent (BOLD) signal responsivity in frontal regions during task‐based fMRI paradigms such as labeling emotional faces (Holzel et al. 2013), sadness provocation (Farb et al. 2010), and self‐referential processing of emotional words (Goldin et al. 2012). Zeidan et al. (2010) reviewed brain mechanisms associated with mindfulness meditation in pain regulation, finding support that both the focused attention and open monitoring aspects of meditation lead to alterations in frontal and other regions during anticipation of pain. However, to date, most fMRI studies of pain regulation have focused on tasks that provoke exteroceptive pain in healthy individuals, rather than tasks involving emotional processing in patients with chronic pain. As an adjunct therapy, one of the goals of MBSR in chronic pain patients may be to address coping with the constant presence of pain, thereby reducing the experiential impact of the pain on one's emotional state, rather than altering the pain itself. Therefore, unlike prior fMRI studies on pain, this study examined the effects of MBSR training on emotional processing, rather than pain responsivity, within patients who have long‐term chronic back pain.

Research regarding the efficacy of MBSR with patient populations may be limited by the time commitment required to participate in the traditional 8‐week course (Kabat‐Zinn 2003). Although few in number, previous studies suggest that abbreviated MBSR courses improve symptoms of depression, anxiety, and quality of life in cancer patients (Stafford et al. 2015), primary care clinicians (Fortney et al. 2013), and an inner city population (Smith et al. 2015). Further, a meta‐analysis in 2009 of all MBSR studies evaluating psychological distress found no relationship between effect sizes of symptom improvement and number of in‐class hours for both clinical and non‐clinical samples (Carmody and Baer 2009). Determining whether an abbreviated MBSR course is effective in reducing symptoms in back pain patients would be of great interest to patients who struggle with availability and clinicians who struggle with resources for such interventions.

We present data from a pilot study aimed to determine whether a 4‐week, abbreviated MBSR training program would improve symptoms in back pain patients, and modulate neural activity in regions associated with the generation and perception of one's emotions. We utilized a previously characterized fMRI task designed to assess awareness of changes in one's current emotional state (Smith et al. 2011, 2014b) to better understand the neural correlates associated with symptom‐relieving effects of MBSR in chronic back pain patients with moderate levels of distress. In a mixed‐models subjects design, based on previous literature, we predicted an abbreviated 4‐week MBSR course would (1) improve depression, anxiety, and back pain symptoms; (2) lead to greater activity within cortical regions implicated in emotion regulation (sgACC, vlPFC, dmPFC, and AI) while gaining awareness to changes in one's emotions; and (3) lead to a correlation between neural activity and verbally reported changes in one's emotional state. This follows from the idea that MBSR may increase conscious (attentional) access to the emotional states. If confirmed, findings would support the conclusion that gaining greater awareness of emotion can have a positive impact on the perception and consequences of chronic back pain.

## Materials and Methods

### Participants and study setting

We recruited 26 right‐handed, chronic low back pain patients (nine males and 17 females) from a spine center offering physical therapy and medical treatments, who were regularly seen by neurorehabilitation physicians. All participants underwent the MRI and completed self‐report measures within 2 weeks before and after the MBSR sessions. Inclusion criteria were being between ages 25 and 60 years old, English speaking, and no evidence of cognitive impairment, other neurological conditions, or psychiatric disorders, other than symptoms of anxiety or depression. We recruited 13 participants for the MBSR group, and 13 participants for the Reading Control (RCon) group. Based on MBSR effects in back pain patients on all three symptoms outcomes of interest to the present study (pain, *d* = 0.69; depression, *d* = 0.78; anxiety, *d* = 0.75; Rosenzweig et al. 2010), a power analysis indicated we would have sufficient power (*β* = 0.80) to detect significant effects with *α* = 0.05. Participants were first given a flyer about a “Research Study on Stress Reduction” and if interested, were interviewed by a member of the research team. Both the flyer and the experimenter described the purpose of the study as, “to see if a popular class called mindfulness‐based stress reduction, or MBSR, helps people with back pain, and what happens in the brain from this treatment.” MBSR was described as involving gentle stretching and greater awareness of the unity of mind and body. Patients were pseudorandomly assigned to MBSR or RCon groups. While complete randomization was intended, study size, timing of the class, and the abbreviated nature of the class made it necessary to assign some participants to the MBSR group based on their ability to attend all four MBSR training sessions, occurring once a week for 4 weeks (i.e., some participants were unable to attend all of the training sessions due to scheduling conflicts and therefore could not be included in the MBSR group).

This study was completed in two waves for the comfort of the MBSR participants in the space available for the training. The first wave included five MBSR participants and eight RCon, the second wave included eight MBSR participants and five RCon. Three participants (one MBSR, two RCon) withdrew from the study prior to undergoing the postintervention assessment. One MBSR and one RCon participant withdrew due to increased back pain and the other RCon participant could not be reached for explanation. We also attempted to contact all participants approximately 1 year after the postintervention assessment to administer surveys evaluating back pain, depression symptoms, and use of any techniques introduced during the study in order to determine whether MBSR training had long‐lasting effects. The participants were not informed beforehand of the 1 year follow‐up assessment; however, approval was obtained by the hospital's Institutional Review Board prior to contact.

The training facilitator was an experienced MBSR teacher who has led patient and professional groups. The abbreviated course met for four training sessions, each 2 h in length, and occurring once a week for 4 weeks. The course covered all techniques taught in the 8‐week course including: following the breath, body scan, guided imagery, loving kindness, mindful eating, mindful walking or other movements, mindful conversations, and observing one's emotions (adapted from Kabat‐Zinn 2003). Mindful walking or other movements were used in place of yoga, which is used in the traditional 8‐week course, in order to modify movements for each person depending on their physical ability (e.g., seated on a chair). The focus, therefore, was on body awareness through movement with breath rather than achieving specific yoga postures. To accommodate the shortened number of sessions, the instructor altered the typical training by (1) introducing more than one topic per class; (2) allowing a shorter period of time devoted to each topic (about 45 min); and (3) allowing less time to practice each technique in the class. The teacher framed the course specifically to the needs and limitations of back pain patients by emphasizing MBSR teachings such as “one is not defined by their pain” and by providing physical modifications to some of the activities as needed. Participants were provided with CDs of MBSR class recordings and had suggested assignments to practice MBSR techniques 20–30 min per day outside of class. The RCon group never met together in person for any training sessions, but rather participants were given a reading material, “Relaxation Techniques for Health: An Introduction” obtained from the National Institutes of Health website (https://nccih.nih.gov/sites/nccam.nih.gov/files/Backgrounder_Relaxation_02-20-2013.pdf?nav=gsa), with no further instructions or demands made upon them to read or practice the material. Topics introduced in the eight‐page reading material included: progressive relaxation, guided imagery, biofeedback, self‐hypnosis, and deep breathing exercises, with selected references to learn more about each topic. All participants were aware that the study was examining the effects of MBSR on back pain and provided written consent which was approved by the hospital's Institutional Review Board.

### Self‐report measures

All participants completed the Beck Depression Inventory‐II (BDI‐II; Beck et al. 1996), the State portion of the State‐Trait Anxiety Inventory (STAI; Spielberger 1983), and the Oswestry Low Back Pain Scale (Oswestry; Fairbank and Pynsent 2000) at preintervention and postintervention MRI appointments.

### Imaging parameters and preprocessing

Participants were scanned at a similar time of day for both scans. On a 3T Philips Ingenia scanner, a gradient echo, echo‐planar pulse sequence was used to exploit the BOLD effect using T2* images. Parameters were as follows: TE = 25 msec, TR = 3000 msec, FOV = 24, 64 × 64 matrix, 40 contiguous axial slices with each slice 4 mm thick. Preprocessing steps were performed using statistical parametric mapping (SPM8, http://www.fil.ion.ucl.ac.uk/spm) implemented in MATLAB 7.12 (MathWorks Inc., Natick, MA). All scans were realigned to correct for motion during tasks, spatially normalized into standardized Montreal Neurological Institute (MNI) anatomical space, and smoothed using an 8 mm^3^ full‐width half‐maximum Gaussian kernel and subsequently high‐pass filtered during first level statistical analyses.

### Sadness induction task

#### Task design

We used a previously validated sadness induction task to focus on neural changes related to becoming aware of one's current emotional state (Smith et al. 2011, 2014b). Briefly (see Smith et al. 2014b for a full description), the participant was instructed to “monitor your emotional state while passively observing the images and listening to the music. Then, indicate by button press when you experience a noticeable change in your mood.” Emotionally congruent visual and auditory stimuli (sad or “neutral”/baseline conditions) were simultaneously presented to aid in the induction of the desired emotion. Task images and music were previously rated for normative sad valence and arousal to confirm efficacy for inducing the desired emotional state (Smith et al. 2014b). The emotionally congruent visual and auditory stimuli were presented until the participant signaled with a button press that they have achieved the desired mood state. This button press initiated a time‐stamped 30 sec “block” of the same stimuli after which the program switched to auditory and visual stimuli to generate the other mood state (sad or neutral) and the process was repeated for the new mood. Alternating sad and neutral mood induction periods were repeated three times each. By using this design, the time to attain a change in mood state is allowed to vary from trial to trial and among individuals in order to ensure that each participant reaches a self‐reportable sad state. “Neutral” was clarified to refer to a relaxed state without any noticeable feeling of sadness. Each participant performed two runs of the task and each run lasted an average of 6.5 min.

#### Task behavioral measures

We recorded the time between initial presentation of sad stimuli and the button press signaling achievement of sad mood (sad mood induction time) and the time between initial presentation of neutral stimuli and the button press signaling achievement of neutral mood (sad mood resolve time) for each participant. After each task run, participants rated sadness intensity and arousal levels for both sad and neutral stimuli on an 8‐point Likert scale. The scale endpoints were “not sad–very sad” and “calm–stimulated”, respectively, with clip art images expressing each emotion (Figure S1).

#### Task analysis

We modeled dynamic changes in emotion using a single regressor for each participant in a fixed effects analysis that shows BOLD correlates in the dmPFC, vlPFC, and AI in a previous report with healthy participants (Smith et al. 2014b). Modeling assumed a monotonic increase from neutral to sad (while viewing sad stimuli) and a similar monotonic decrease from sad to neutral (while subsequently viewing neutral stimuli). Equal increments from 0 to 1 were used to model the increase from the start of the presentation of sad stimuli (after participants first confirmed they felt neutral) until the button press indicating that the participant had achieved a sad state, and increments from 1 to 0 were used to model the decrease from sad to neutral, as done previously (Smith et al. 2014b). Modeling the dynamic emotional changes, rather than the 30 sec blocks of stable emotion, was chosen to best assess the theory that MBSR's positive impact on back pain may be related to increased awareness of one's own emotional state. A fixed effects analysis of each participant's time series was used for second order group analyses.

### Control vision BOLD task

In our general study design, we included a short vision fMRI task that was hypothesized to be stable over time and unaffected by the intervention. This short task shows strong BOLD signal in the visual cortex. The task consists of viewing full color pictures comprising outdoor scenes with at least five natural or artificial objects (e.g., a scene of houses on the edge of the ocean) alternated with a 26‐size‐font white crosshair on a black background, as a baseline, for 30 sec in each block (10 pictures per block), for a total scan time of 2 min. A contrast map for each individual representing greater BOLD response during the scenes vs. baseline was generated for second order group analyses. All participants received this Control Vision Task first in the scanner, before the Sadness Induction Task, and there is no evidence that the Control Vision Task would influence findings on the Sadness Induction Task.

### Statistical analyses

We evaluated both the interactive effect of group on dependent measures of interest via ANOVA, as well as within group pre‐ to post‐intervention changes over time via paired *t*‐tests. Given the small sample size, we used Fisher's protected *t*‐test with planned contrast, based on a priori predictions, in order to minimize both Type I and II errors and control for family‐wise error (Cohen et al. 2003). Behavioral dependent measures were: (1) summary scores of depression, anxiety, and back pain; and (2) mean sad induction/resolve time and stimuli valence/arousal ratings for the sad induction fMRI tasks. For fMRI data, the MARSBAR tool (http://marsbar.sourceforge.net/) was used within SPM8 to extract mean BOLD signal values from four bilateral regions of interest (ROIs). These ROIs included sgACC (BA25), AI, dmPFC, and vlPFC (Fig. 2A). The ROIs were selected because these regions showed a significant association with this sadness induction task in our previous studies (Smith et al. 2011, 2014b) and because of the contributions of these regions to afferent and efferent emotional/autonomic processing, as discussed in detail elsewhere (Smith et al. 2014a). The sgACC and AI ROIs have been used previously by our group and are described elsewhere (Lane et al. 2013; Smith et al. 2014a). The dmPFC and vlPFC ROIs were created with the AAL WFUPickAtlas tool (Winston‐Salem, NC) used within SPM8. For the Control fMRI task, left and right visual cortex ROIs were generated in WFUPickAtlas. In fitting with intention‐to‐treat (ITT) concept, participants remained in the groups to which they were assigned, regardless of their adherence to the treatment or subsequent withdrawal from the study (Gupta 2011). Exploratory *t*‐tests were conducted between groups on preintervention means to determine any baseline group differences. For all analyses alpha was set at *P *<* *0.05.

## Results

### Participants and survey measures

Table 1 summarizes group demographic variables and survey measures. No between‐group differences in age [*t*(21) = 0.62; *P *=* *0.54] or years of education [*t*(21) = 0.08; *P *=* *0.94] were observed. Groups were well balanced for gender and medication use to manage pain and psychiatric symptoms (see Table S1 for complete medication list by the participant). Of the RCon group, seven participants reported reading the material. As expected, there were consistently more participants from the MBSR group who did additional MBSR research outside of what was provided in the study and used the techniques they learned than the RCon group. MBSR participants also used the techniques at a higher frequency than the RCon group. Groups were, however, balanced on positive life changes made through the course of the study, such as diet, exercise, and counseling.

**Table 1:** Group demographic variables and survey measures

	MBSR (*n* = 12)	RCon (*n* = 11)
Age (mean years ± SD)	46.0 ± 11.3	43.0 ± 2.5
Education (mean years ± SD)	14.5 ± 2.5	14.5 ± 2.8
Gender (female/male)	8F/4M	6F/5M
Pain medication	*n* = 5	*n* = 4
Psychiatric medication	*n* = 4	*n* = 3
Read control material	n/a	*n* = 7
Additional MBSR research	*n* = 5	*n* = 3
Used MBSR techniques	*n* = 10	*n* = 6
Average frequency of use	2×/week	<1×/week
Positive life changes (e.g. diet, exercise, counseling)	*n* = 5	*n* = 5

	Pre	Post	Pre	Post
BDI‐II: Depression symptoms (mean score ± SD)	16.8 ± 11.7	11.4 ± 9.2*	12.2 ± 9.8	9.3 ± 8.1*
BDI‐II: Somatic‐Affective Subscale (mean score ± SD)	9.4 ± 5.0	6.6 ± 5.1*	7.1 ± 6.0	6.0 ± 5.3
BDI‐II: Cognitive Subscale (mean score ± SD)	5.7 ± 6.2	3.8 ± 4.1#	3.8 ± 3.7	2.2 ± 2.9*
Oswestry (mean score ± SD)	18.9 ± 8.3	16.6 ± 9.0*	15.4 ± 9.2	13.8 ± 9.4
STAI (mean score ± SD)	39.0 ± 12.3	38.8 ± 13.2	29.0 ± 7.7	31.7 ± 10.8

*P <* *0.05; ^#^ *P *<* *0.10 vs. preintervention.

MBSR, mindfulness based stress reduction; RCon, Reading control; BDI‐II, Beck Depression Inventory‐II; STAI, State‐Trait Anxiety Inventory; SD, standard deviation.

**Figure 1:**
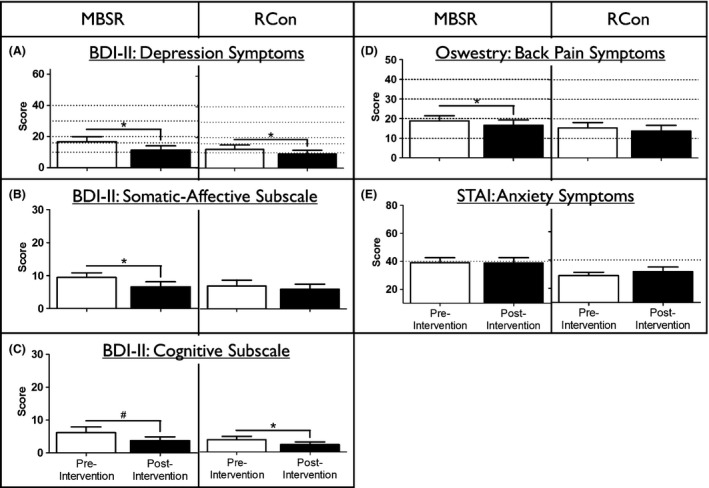
Pre‐ and post‐intervention survey measure means (±SE) presented by group with dashed lines representing clinical norms. (A) Beck Depression Inventory‐II (BDI‐II): Preintervention, the mindfulness‐based stress reduction (MBSR) group scored in the “borderline clinical depression” category, and the Reading control (RCon) group scored in the “mild mood disturbance” category. Postintervention, both groups improved, dropping the MBSR group into the “mild mood disturbance” category and the RCon group to the “normal disturbances” category. Categories based on a five‐level severity system. (B) BDI‐II Somatic‐Affective Subscale: The MBSR group showed significant improvement. (C) BDI‐II Cognitive Subscale: The MBSR group showed a trend towards improvement and the RCon group showed significant improvement. (D) Oswestry Low Back Pain Disability Questionnaire: Preintervention both groups scored in the “moderate disability” category. Postintervention the MBSR group significantly improved but both groups remained in the “moderate disability” category. (E) State‐Trait Anxiety Inventory (STAI): PreIntervention, both groups scored below clinically significant symptoms for state anxiety; the MBSR group reported a higher mean level of anxiety than the RCon group (*P *=* *0.03). Postintervention, neither group showed changes in state anxiety. *P <* *0.05; ^#^ *P *<* *0.10.

The MBSR group reported somewhat higher levels of depression, anxiety, and back pain, although only the between‐group anxiety difference reached statistical significance [STAI: *t*(21) = 5.34; *P *=* *0.031; Fig. 1E]. Preintervention, both groups showed elevated depression symptoms based on a five‐category severity system; the mean BDI‐II score placed the MBSR group as “borderline clinical depression” while the RCon mean score was in the “mild mood disturbance” category (Kress et al. 2003). Mean scores on the Oswestry self‐report of back pain placed both groups in the “moderate disability” category. For anxiety, both group's mean scores were below the clinically significant cutoff mark of 40 although the MBSR group had a mean score of 39, representing borderline clinical significance (Julian 2011). There were no interactive effects of group; therefore, all subsequent analyses reflect within‐group changes pre‐ to post‐intervention. Only the MBSR group significantly improved in self‐reported back pain [Oswestry; *t*(9) = 2.30; *P *=* *0.04; *d *=* *0.28; Fig. 1D]. Both the MBSR and the RCon groups improved in total depression symptoms [BDI‐II; MBSR: *t*(11) = 2.30; *P *=* *0.04; *d *=* *0.58; RCon: *t*(10) = 2.27; *P *=* *0.047); *d *=* *0.36; Fig. 1A]. When the BDI‐II subscales were examined (Steer et al. 1999), the MBSR group showed significant improvement in the Somatic‐Affective Subscale [*t*(11) = 2.61; *P *=* *0.024; *d *=* *0.57; Fig. 1B], and a trend towards improvement in the Cognitive Subscale [*t*(11) = 2.04; *P *=* *0.066; *d *=* *0.60; Fig. 1C]. The RCon group significantly improved in the Cognitive Subscale [*t*(10) = 2.75; *P *=* *0.02; *d *=* *0.53; Fig. 1C] but not the Somatic‐Affective Subscale [Fig. 1B]. Neither group significantly improved in anxiety symptoms [STAI; *P* = ns; Fig. 1E]. As three participants withdrew from the study, ITT analysis was carried out by using the last observation carried forward method (Gupta 2011), yielding no change in the statistical significance in any outcome measure for either group.

Postintervention, the MBSR mean BDI‐II score fell into the “mild mood disturbance” category, and the RCon mean was within the “normal disturbances” category. Despite improvement after MBSR training, both treatment and control groups remained in the “moderate disability” category according to Oswestry scores (see Table S2 for number of participants within each symptom category pre‐ and post‐intervention).

### fMRI

#### Sadness induction task behavior

Table 2 summarizes sad mood induction/resolve times (2a) and valence/arousal ratings for sad and neutral stimuli (2b). Time and ratings did not significantly change within groups from pre‐ to post‐intervention nor differ between‐groups at the preintervention scan. As expected, sad stimuli were rated as more sad and arousing than neutral stimuli preintervention [Valence: *t*(22) = 8.47; *P *<* *0.0001; Arousal: *t*(22) = 3.54; *P *=* *0.002] and postintervention [Valence: *t*(22) = 7.90; *P *<* *0.0001; Arousal: *t*(22) = 3.29; *P *=* *0.003] across all participants.

**Table 2:** Sadness Induction Task Behavior. (a) Mean (±SD) time (sec) for participants to induce and resolve sad mood. (b) Mean Likert scale ratings (±SD) of sad and neutral images

(a)				
	Preintervention		Postintervention	
	Sad induction	Sad resolve	Sad induction	Sad resolve
MBSR	30.1 (±18.0)	30.7 (±17.2)	29.8 (±25.4)	31.3 (±16.4)
RCon	42.0 (±20.7)	43.0 (±30.4)	45.3 (±22.8)	37.4 (±23.0)

(b)					
	Images	Preintervention		Postintervention	
		Sadness intensity	Arousal	Sadness intensity	Arousal
MBSR	Sad	5.5 (±1.4)	5.4 (±1.7)	5.5 (±2.0)	5.3 (±1.8)
	Neutral	2.0 (±1.2)	2.9 (±1.4)	1.25 (±0.5)	2.7 (±1.6)
RCon	Sad	5.2 (±2.6)	3.8 (±2.4)	4.7 (±1.6)	4.2 (±1.9)
	Neutral	1.5 (±0.9)	2.8 (±2.3)	2.0 (±1.2)	3.2 (±2.1)

MBSR, mindfulness‐based stress reduction, RCon, Reading control; SD, standard deviation.

#### Sadness induction BOLD response

**Figure 2:**
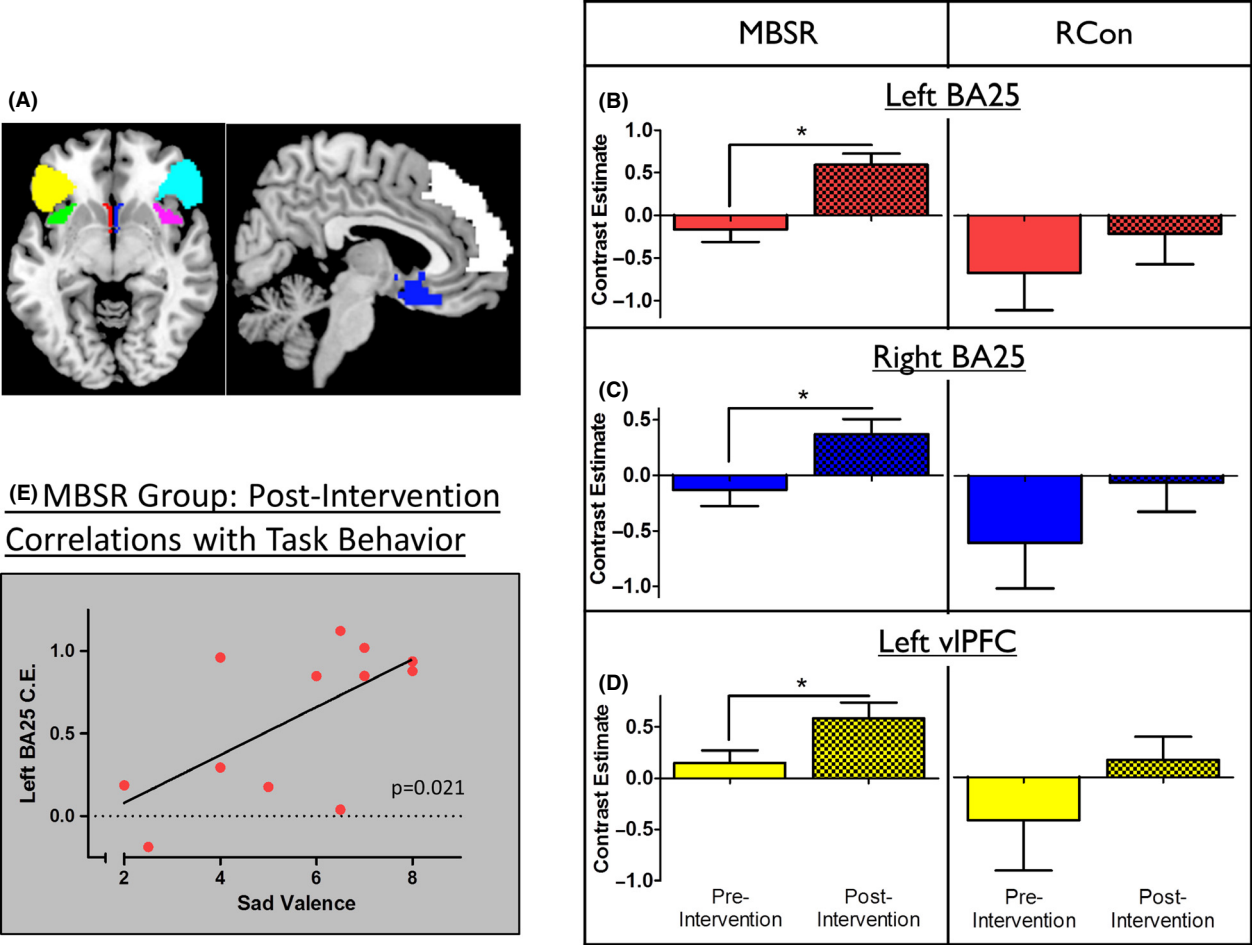
Sadness induction task blood oxygen‐level‐dependent signal changes: (A) Regions of interest template: ventrolateral prefrontal cortices (vlPFC) left (yellow) and right (teal); anterior insula left (green) and right (magenta); subgenual anterior cingulate cortex (sgACC) left (red) and right (blue); dorsomedial prefrontal cortices left (data not shown) and right (white). The mindfulness‐based stress reduction (MBSR) group showed significant increases in left sgACC (B), right sgACC (C), and left vlPFC (D) from pre‐ to post‐intervention. (E) In the MBSR group, postintervention activation in left sgACC positively correlated with sad intensity ratings of task images used to induce sad mood. *P <* *0.05.

Preintervention, there were no significant between‐group differences in BOLD signal. There were also no interactive effects of group; therefore, all subsequent analyses reflect within‐group changes pre‐ to post‐intervention. As predicted, only the MBSR group showed BOLD signal changes, with significant increases in the left sgACC [*t*(11) = 20.37; *P *=* *0.0009; *d *=* *1.68; peak voxel SPM(*t*) = 7.41, MNI: ‐6 12 ‐14, cluster size: 74 voxels; Fig. 2B], right sgACC [*t*(11) = 7.53; *P *=* *0.019; *d *=* *1.06; peak voxel SPM(*t*) = 3.47, MNI: 2 14 ‐12, cluster size: 39 voxels; Fig. 2C], and left vlPFC [*t*(11) = 4.95; *P *=* *0.032; *d *=* *1.04; peak voxel SPM(*t*) = 3.87, MNI: ‐48 24 ‐12, cluster size: 52 voxels; Fig. 2D]. There were also trend level increases in the MBSR group in the left dmPFC [*t*(11) = 3.74; *P *=* *0.079; *d *=* *1.26], right dmPFC [*t*(11) = 3.37; *P *=* *0.094; *d *=* *1.27], and right vlPFC [*t*(11) =3.75; *P *=* *0.079; *d *=* *0.72]. Postintervention, within the MBSR group only, BOLD signal in the left sgACC positively correlated with sad valence ratings [*r*(10) = 0.65, *P *=* *0.021; Fig. 2E] and signal in the right sgACC positively correlated with sad valence at a level approaching significance [*r*(10) = 0.56, *P *=* *0.057].

#### Control task BOLD response

On the “control” task, strong BOLD signal in the visual cortex was observed with no interactive effects of group or within‐group changes pre‐ to post‐intervention. This suggests that changes in MBSR signal associated with awareness to changes in one's current emotional state are not due to general differences in BOLD responsivity.

### One year follow‐up

Approximately 1 year after the postintervention assessment, 15 participants (MBSR, *n* = 9; RCon, *n* = 6) were reached for a brief telephone follow‐up focusing on BDI‐II and Oswestry assessments. The MBSR group did not maintain significant within‐group improvement on the Oswestry (PreIntervention: 19, ±9.3; Follow‐up: 17.5, ±9.2; *P *=* *0.54) and neither group maintained significant within‐group improvement on the BDI‐II (MBSR: PreIntervention: 16.1, ±10.6; Follow‐up: 14.3, ±9.1; *P *=* *0.62; RCon: PreIntervention: 11, ±8.6; Follow‐up: 11.5, ±15.7; *P *=* *0.92).

## Discussion

The present pilot study found that an abbreviated 4‐week MBSR course had significant effects on pain symptoms and somatic‐affective aspects of depression in back pain patients. We also found associated changes in activity within neural networks implicated in emotional processing, which suggested that these patients attained greater attentional access to their emotional states. These findings jointly suggest that increased awareness of one's own emotions may positively impact back pain symptoms; however, more research is necessary to confirm this hypothesis. Interestingly, this short course of MBSR did not produce a unique effect on cognitive aspects of depression or anxiety symptoms, which suggests that there may be a cumulative dose–response aspect to MBSR intervention such that neural changes and coping with pain occur prior to changes in more enduring symptoms such as cognitive aspects of depression and anxiety, or that the intense focus on pain in these patients made changes to this symptom more evident.

### Improvement in symptoms

The MBSR intervention led to unique improvements in back pain symptoms, as evident in the significant improvement in the MBSR group only. Of further interest is whether improvements in back pain were clinically significant. It is suggested that a clinically significant improvement is observed with a 4+ point change on the Oswestry (Roland and Fairbank 2000). Half of the MBSR participants reached this threshold of improvement, while only 18% reached this level of improvement in the control group. The same pattern was observed for the BDI‐II, with more (42%) MBSR participants attaining a 50% reduction in depression symptoms while only 18% reached this level of improvement in the control group. Group comparison of the BDI‐II subscales showed that the MBSR group significantly improved on the Somatic‐Affective Subscale and total depression symptoms; however, the RCon groups also significantly improved in total depression symptoms and cognitive aspects of depression suggesting a regression to the mean, beneficial effect of the reading intervention, and/or beneficial effects of the inherent self‐reflection involved in self‐report mood studies. In support of the last, anecdotally, several control participants commented that they had been compelled by the self‐reflective experience during the baseline visit to seek out emotional help on their own.

Our findings are consistent with others who have shown favorable outcomes of traditional 8‐week courses on pain severity (Morone et al. 2008; Rosenzweig et al. 2010) and depression in back pain patients (Rosenzweig et al. 2010; Schutze et al. 2014), and other patient populations (Lengacher et al. 2009; Goldin and Gross 2010; Joo et al. 2010; Kimbrough et al. 2010; Vollestad et al. 2011; Asmaee Majid et al. 2012; Omidi et al. 2013; Johns et al. 2015; Serpa et al. 2014). Although MBSR has been effective in alleviating symptoms in anxiety disorders, results regarding the reduction of anxiety in back pain patients have been mixed.; Schutze et al. (2014) reported no change, but Rosenzweig et al. (2010) reported reduced anxiety. The unique improvements in pain and somatic‐affective aspects of depression observed with the MBSR intervention may be related to an increased sense of empowerment, which can be particularly important for chronic pain patients (Werner and Malterud 2005). The MBSR instructor attempted to empower patients through teaching techniques that would enable the participants to choose their response to a given experience, specifically related to not being defined by pain. This follows from the core MBSR teaching of separating oneself from habitual patterns of response, observing one's experience, and then choosing how to respond.

### An abbreviated MBSR program

To our knowledge, this is the first study to compare an abbreviated MBSR intervention to a control group in a clinical population. Our results, taken together with studies of various lengths of MBSR training, suggest a potential dose–response in symptom improvement for back pain patients. MBSR‐related improvements in perceived pain seem to be sensitive to a low‐dose MBSR intervention, whereas a unique improvement in cognitive aspects of depression and anxiety may require a larger dose of MBSR, as has been seen in the traditional course (Morone et al. 2008; Rosenzweig et al. 2010; Schutze et al. 2014). The 8‐week intervention has shown long‐term benefits in other patient populations (Grossman et al. 2007), but we failed to find long‐term benefits of the 4‐week intervention on pain and depression symptoms in our sample of back pain patients. Indeed, even the original MBSR program is arguably a short intervention, meant to be a catalyst for a more enduring change in life style. In most of our participants, this short intervention did not produce such enduring changes. Our findings of improved symptoms and neural changes associated with current use suggest that the mechanism by which MBSR changes perceived pain is quickly acquired, but also likely linked to a state effect of increased awareness, such that longer exposure to MBSR techniques provides a foundation that is necessary for long‐term benefits of symptom improvements.

### MBSR effects on neural correlates of emotional awareness

Previous studies of the neural substrates associated with pain perception have utilized pain paradigms to determine whether MBSR alters pain tolerance as elicited by either heat, cold, or electrical stimulation (Zeidan et al. 2010). These past studies therefore focus on the anticipatory aspects of pain regulation, and Zeidan et al. (2010) hypothesized that MBSR plays a role in altering expectancy of pain by increasing the training of “reduced‐ or non‐appraisal of nociceptive information.” We approached the question of mechanism from a different vantage point by using an emotional processing paradigm that probes frontal regions implicated in generating and consciously accessing one's own emotions. We found that MBSR intervention uniquely led to greater activity of the sgACC and vlPFC while gaining conscious access to changes in emotion, as well as a greater relationship between activity in sgACC (associated with generating emotional reactions) and changes in self‐reported emotional states. The sgACC has been implicated in the representation of emotional valence (Grimm et al. 2006), appraising the emotional significance of one's current situation (Roy et al. 2012), and automatically generating emotional “somatic marker” responses to guide decision‐making (Gupta et al. 2011). The relationship between sgACC and emotional regulation is complex; in patients with severe depression, sgACC hypermetabolism is common, and activation in this area normalizes with alleviation of depression through successful therapies (Goldapple et al. 2004; Mayberg et al. 2005; Price and Drevets 2012). In our study, after MBSR training, activation in sgACC positively correlated with sadness intensity ratings. As sadness intensity ratings did not change from pre‐ to post‐intervention in the MBSR group, our findings suggest that MBSR induced an increased coupling of self‐reported emotional experience and the neural activity of sgACC associated with emotion generation. That is, this finding suggests that participants had greater conscious access to their emotions after they were generated. Others have reported this phenomenon, noting that in healthy participants increased blood flow in sgACC was coupled with self‐reported transient sadness (Mayberg et al. 1999). Consistent with this finding, Farb et al. (2010) also found MBSR training to be related to increased involvement of sgACC when depressed and anxious patients observed sad movie clips. Holzel et al. (2013) found a similar increased coupling of vlPFC in generalized anxiety disorder patients whose anxiety was alleviated by MBSR. This is interesting, as MBSR training also led to an increased engagement of left vlPFC while gaining awareness to changes in one's emotional state in this study, and activity in this region was associated with becoming aware of sadness in our previous study with healthy adults (Smith et al. 2014b). Taken together, findings from this study converge with conclusions from a recent meta‐analysis and review, that short‐term mindfulness training is associated with increased “top‐down” emotion regulation, while increased “bottom‐up” emotion regulation is more likely to be observed in long‐term practitioners (Chiesa et al. 2013; Tang et al. 2015).

From a therapeutic standpoint, MBSR focuses on increasing awareness of present moment experiences (Kabat‐Zinn 1990); therefore, increased coupling between sgACC activity and self‐reported emotional transitions may relate to core aspects of MBSR training. Using magnetoencephalography, Kerr et al. (2013) found that the introductory technique of MBSR, the body scan, trains one to control the “attentional spotlight” within the somatosensory cortex, and postulated that over time, this attentional control may become generalized to other cognitive processes subserved by regions of the prefrontal cortex. The attentional control gained through the body scan may be particularly relevant to chronic pain patients by refocusing pain to a specific body region, rather than as an overwhelming feeling of generalized pain, which in turn may “free up pain‐focused attentional resources” (Kerr et al. 2013). Our findings support this hypothesis in that back pain patients who received MBSR training showed reductions in perceived pain and a concomitant increase in regional prefrontal neural activity reflecting a gain in cognitive access to one's own emotional state.

### Study limitations

Interpretations from this pilot study are limited by the sample size and the pseudorandom design that was necessary to ensure that all MBSR participants were able to attend sufficient sessions in this abbreviated program. Although the present pilot study had sufficient power to detect a pattern of statistically significant changes across behavioral and neural modalities associated with abbreviated MBSR treatment, these findings should be replicated in future larger studies powered to detect a significant interaction between MBSR and control groups. This pseudorandom design resulted in a possible selection bias where, in a small percentage of cases, less motivated individuals (i.e., unwilling to clear their schedule) were more likely to be assigned to the control group. The choice to proceed with the pseudorandom design presented a possible confound for interpretation, but was necessary due to budgetary restrictions and the need to evaluate all participants at the same time in order to avoid cohort effects (i.e., holidays, seasons, etc.). Although groups were not statistically significantly different in preintervention analyses for back pain, depression symptoms, or neuroimaging measures, the groups do appear somewhat dissimilar, which is a possible problem one can encounter with a small sample. The purpose of this pilot study was to provide proof of concept for the feasibility of an abbreviated MBSR course and lay the ground work for a larger, fully randomized trial.

Additionally, choice of control group for behavior interventional studies is challenging; the inherent nature makes blinding and placebo controls difficult. The MBSR group likely had expectation to improve in symptoms. Further, the control group was aware of the fact that they were being compared to an in‐person MBSR training group, which may have created the expectation that, by comparison, they would experience worsening symptoms. In order to mitigate this expectation effect, the stress reduction reading material was administered to the control group as an alternative treatment. The specific reading materials were obtained from an NIH website in order to compare MBSR intervention to materials which are freely available to everyone, with the benefit of reflecting current real‐world options provided to patients in our spine center. As recommended by Tang et al. (2015), the present pilot study's longitudinal design with an active control group has arguably more utility than cross‐sectional designs and waitlist control groups commonly used. Perhaps the most rigorous comparison group, however, should control for social interactions associated with the group and teacher (Morone et al. 2009; Tang et al. 2015). A third group with no intervention may be further useful in interpreting the “regression to the mean” phenomenon sometimes observed in chronic pain studies. Future studies investigating brain and behavior changes associated with an abbreviated MBSR course are warranted to address the limitations of this pilot study.

In this pilot study, we did not match participants on demographic variables, control for medication use, or control for other changes in behavior that participants engaged in during the study. Demographic variables and medication use, however, were strikingly equivalent between groups (Table 1). Further, adding any of these demographic variables as a covariate into within‐subjects repeated measures ANCOVA did not change the effect of MBSR or RCon interventions on any outcome measure. In an exit survey, we also learned the extent to which participants interacted with reading control materials, did additional MBSR research, used the techniques learned, and made positive changes in their lives in other areas such as diet, exercise, and counseling. Reassignment of groups based on any of these factors did not better account for improvements in symptoms than participation in the MBSR group, making it unlikely that there were other mediating variables in symptom improvement over and above MBSR. Lastly, while a correlation between changes in neural activity and changes back pain pre‐ to post‐MBSR intervention would provide a strong basis for changes in emotional awareness as being related to symptom improvement, this correlation was not statistically significant. MBSR participants were very consistent in their back pain improvement, thus the restricted range and small sample size were not conducive to detecting a significant correlation. Future larger studies are warranted to assess this relationship.

## Conclusion

Our pilot study suggests that abbreviated MBSR is an effective complementary intervention for back pain patients. Further, our fMRI analyses suggest that MBSR's positive impact on back pain may be related to increased awareness of one's own emotional state; however, more research is necessary to confirm this hypothesis. Although some state‐dependent changes in symptoms and neural functioning can be observed in the short program, the 8‐week intervention may provide a more firm foundation for deeper emotional changes in anxiety and cognitive aspects of depression that are maintained for a longer period of time. Ideally, health programs could teach MBSR on a weekly basis, allowing patients like those with chronic pain who find it difficult at times to travel, more flexibility to gain MBSR skills over a longer period of time. Determining the dose response of benefits associated with MBSR is of great value for patients who struggle with availability and clinicians who struggle with resources for such interventions. Further, understanding the neural processes by which MBSR exerts beneficial effects adds valuable information to the choice of appropriate patient populations for this intervention.

## Conflict of Interest

None declared.

## Supporting information


**Figure S1**. How do you feel right now?

**Table S1**. Absolute medication use by group and participant.
**Table S2**. Number of participants in each five‐category severity ranking by group, pre‐ and post‐intervention.
