# Supplementary figures and images for: Brain and behavior changes associated with an abbreviated 4‐week mindfulness‐based stress reduction course in back pain patients
Source: Brain Behav. 2016 Feb 16;6(3):e00443. doi: 10.1002/brb3.443 (PMC4754498; doi:10.1002/brb3.443)

How do you feel right now?

Valence

Not sad

Very sad

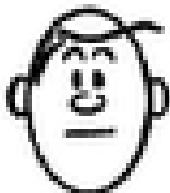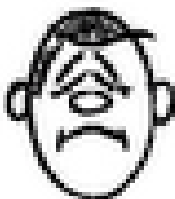

1 2 3 4 5 6 7 8

Arousal

Calm

Stimulated

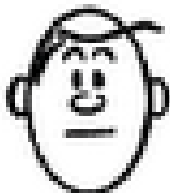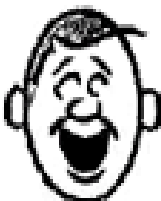

1 2 3 4 5 6 7 8

Supplement: Supplementary file 1 — Figure S1. How do you feel right now? [file BRB3-6-e00443-s001.pdf]
