# Supplementary material for: Brain and behavior changes associated with an abbreviated 4‐week mindfulness‐based stress reduction course in back pain patients
Source: Brain Behav. 2016 Feb 16;6(3):e00443. doi: 10.1002/brb3.443 (PMC4754498; doi:10.1002/brb3.443)
Supplement: Supplementary file 2 — Table S1. Absolute medication use by group and participant. Table S2. Number of participants in each five‐category severity ranking by group, pre‐ and post‐intervention. [file BRB3-6-e00443-s002.docx]

**Appendix**

Supplemental Table 1. Absolute medication use by group and participant.

| Group | Subject | Medications |
| --- | --- | --- |
| MBSR | Subject1 | Oxycodone, Oxycontin, Albuterol, Diazepam |
|  | Subject2 | Oxycodone 5mg, Androgel 1.0%, Linsinopril, Mirtazapine |
|  | Subject3 | Trazodone, Butinol |
|  | Subject4 | Flecainide, Sloniacin, Pravastatin |
|  | Subject5 | N/A |
|  | Subject6 | N/A |
|  | Subject7 | N/A |
|  | Subject8 | Prozac, Klonopin, Norco, Flexol |
|  | Subject9 | Oxycodone, Tylenol, Metformin, Simvastatin, Lisinopril |
|  | Subject10 | N/A |
|  | Subject11 | N/A |
|  | Subject12 | N/A |
| RCon | Subject13 | Morphine Sulfate, Baclofen |
|  | Subject14 | Percocet, Lexepro, Lisinopril, Humira, Proventil, Duo-neb, Aspirin, Insulin Pen, Soma, Cymbalta, Advair, Neurontin, Hydrodiuril, Lantus Solostar, Xalatan, Prinivil, Glucophage, Methotrexate, Proair, Zocor, Trezix, Hytrin, Desyrel, Levitra |
|  | Subject15 | N/A |
|  | Subject16 | Simvastatin |
|  | Subject17 | N/A |
|  | Subject18 | Trazodone, Flexeril, Vicodin |
|  | Subject19 | Trazodone, Maloxicam, Codeine, Fluoxetine |
|  | Subject20 | N/A |
|  | Subject21 | N/A |
|  | Subject22 | N/A |
|  | Subject23 | N/A |

Supplemental Table 2. Number of participants in each 5-category severity ranking by group, pre- and post-intervention.

|  | | MBSR | | RCon | |
| --- | --- | --- | --- | --- | --- |
|  |  | Pre-Intervention | Post-Intervention | Pre-Intervention | Post-Intervention |
| BDI-II | Normal | 4 | 5 | 5 | 7 |
|  | Mild Mood Disturbances | 4 | 3 | 1 | 0 |
|  | Borderline Clinical Depression | 0 | 2 | 3 | 3 |
|  | Moderate Depression | 2 | 2 | 2 | 1 |
|  | Severe Depression | 2 | 0 | 0 | 0 |
| Oswestry | Minimal Disability | 2 | 3 | 3 | 5 |
|  | Moderate Disability | 4 | 2 | 5 | 4 |
|  | Severe Disability | 3 | 5 | 2 | 2 |
|  | Crippled | 1 | 0 | 1 | 0 |
